# 2-[(2,4-Dihydroxy­benzyl­idene)amino]-3′,6′-bis­(ethyl­amino)spiro­[isoindoline-1,9′-xanthen]-3-one

**DOI:** 10.1107/S1600536810015126

**Published:** 2010-05-29

**Authors:** Zhi-Hong Xu, Yan-Ling Zhang, Yan-Ru Zhao, Feng-Ling Yang

**Affiliations:** a College of Chemistry and Chemical Engineering, Xuchang University, Xuchang, Henan Province 461000, People’s Republic of China

## Abstract

The title compound, C_35_H_36_N_4_O_4_, was prepared as a spiro­lactam ring formation of rhodamine B dye for comparison with a ring-opened form. The xanthene ring system is approximately planar. The r.m.s. deviation from planarity is 0.064 (6) Å for the xanthene ring. The dihedral angles formed by the spiro­lactam and 2,4-dihydroxy­benzene rings with the xanthene ring system are 86.6 (9) and 88.0 (9)°, respectively.

## Related literature

For the structures of rhodamine derivatives bearing a lactam moiety, see: Deng *et al.* (2009 ▶); Kwon *et al.* (2005 ▶); Tian & Peng (2008 ▶); Wu *et al.* (2007 ▶); Xu *et al.* (2009 ▶); Zhang *et al.* (2008 ▶).
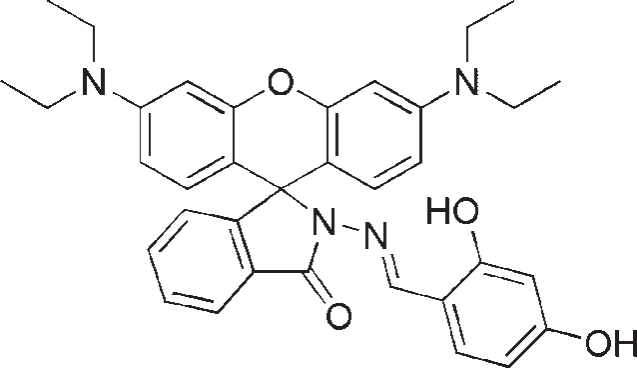


## Experimental

### 

#### Crystal data

C_35_H_36_N_4_O_4_
*M* *_r_* = 576.68
Monoclinic, 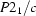

*a* = 9.4461 (4) Å
*b* = 26.6905 (12) Å
*c* = 12.2453 (5) Å
β = 104.423 (2)°
*V* = 2990.0 (2) Å^3^
*Z* = 4
Mo *K*α radiation
μ = 0.09 mm^−1^
*T* = 296 K
0.25 × 0.23 × 0.21 mm

#### Data collection

Bruker APEXII CCD diffractometer
Absorption correction: multi-scan (*SADABS*; Sheldrick, 1996 ▶) *T*_min_ = 0.979, *T*_max_ = 0.982
15630 measured reflections
5310 independent reflections
2162 reflections with *I* > 2σ(*I*)
*R*_int_ = 0.087

#### Refinement

*R*[*F* ^2^ > 2σ(*F* ^2^)] = 0.070
*wR*(*F* ^2^) = 0.215
*S* = 1.02
5310 reflections
393 parameters
H-atom parameters constrained
Δρ_max_ = 0.25 e Å^−3^
Δρ_min_ = −0.22 e Å^−3^

### 

Data collection: *APEX2* (Bruker, 2005 ▶); cell refinement: *SAINT* (Bruker, 1998 ▶); data reduction: *SAINT*; program(s) used to solve structure: *SHELXS97* (Sheldrick, 2008 ▶); program(s) used to refine structure: *SHELXL97* (Sheldrick, 2008 ▶); molecular graphics: *SHELXTL* (Sheldrick, 2008 ▶); software used to prepare material for publication: *SHELXTL*.

## Supplementary Material

Crystal structure: contains datablocks I, global. DOI: 10.1107/S1600536810015126/jh2147sup1.cif

Structure factors: contains datablocks I. DOI: 10.1107/S1600536810015126/jh2147Isup2.hkl

Additional supplementary materials:  crystallographic information; 3D view; checkCIF report

## Figures and Tables

**Fig. 1.**
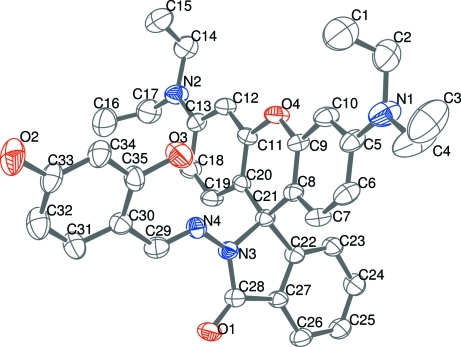
The molecular structure of the title compound, with displacement ellipsoids drawn at 30% probability level.

## supplementary crystallographic information

### Comment

Among many fluorescent compounds, rhodamine dyes are known to have excellent photophysical properties, and they are one of the most widely used fluorophores for labeling and sensing biomolecules. There are a few single-crystal reports about rhodamine derivatives bearing a lactam moiety (Xu *et al.*, 2009; Kwon *et al.*, 2005; Wu *et al.*, 2007; Zhang *et al.*, 2008; Tian *et al.*, 2008; Deng *et al.*, 2009). Detailed information on their molecular and crystal structures is necessary to understand their photophysical and photochemical properties.

In agreement with other reported models , (Xu *et al.*, 2009; Wu *et al.*, 2007; Zhang *et al.*, 2008; Tian *et al.*, 2008;) the main skeleton of the molecule is formed by the xanthene ring and the spirolactam-ring. As shown in Figure 1, The atoms of the xanthene ring or the spirolactam-ring are both nearly planar and are almost perpendicular to each other. The dihedral angle between the xanthene mean planes and the spirolactam ring fragment is 86.6 (9)°. The dihedral angle between the xanthene mean planes and the 2,4-dihydroxybenzene ring is 88.0 (9)°.

### Experimental

A portion of rhodamine B hydrazide (0.46 g, 1 mmol) and 2,4-dihydroxybenzaldehyde (0.16 g, 1.2 mmol) were mixed in 20 ml e thanol and three drops HAc was added. The reaction solution was refluxed for 3 hours under N_2_ atmosphere, the reslulting solution was evaporated to 10 ml and allowed to stand at room temperature overnight. The reddish crystals which appeared next day were filtered and washed by ethanol to give 0.46 g of the title compound in 80% yield. Single crystals suitable for X-ray measurements were obtained from mother liquid by slow evaporation at room temperature.

### Refinement

The H atoms attached to C, N and O atoms were placed in geometrically calculated positions (C—H = 0.93–0.97 Å and O—H = 0.82 Å) and refined as riding, with *U*_iso_(H) = 1.2*U*_eq_(C, N) or 1.5*U*_eq_(methyl C, O).

### Crystal data

**Table:**

C_35_H_36_N_4_O_4_	*F*(000) = 1224
*M**_r_* = 576.68	*D*_x_ = 1.281 Mg m^−^^3^*D*_m_ = 1.281 Mg m^−^^3^*D*_m_ measured by not measured
Monoclinic, *P*2_1_/*c*	Mo *K*α radiation, λ = 0.71073 Å
*a* = 9.4461 (4) Å	Cell parameters from 922 reflections
*b* = 26.6905 (12) Å	θ = 2.3–17.5°
*c* = 12.2453 (5) Å	µ = 0.09 mm^−^^1^
β = 104.423 (2)°	*T* = 296 K
*V* = 2990.0 (2) Å^3^	Block, colorless
*Z* = 4	0.25 × 0.23 × 0.21 mm

### Data collection

**Table:**

Bruker APEXII CCD diffractometer	5310 independent reflections
Radiation source: fine-focus sealed tube	2162 reflections with *I* > 2σ(*I*)
graphite	*R*_int_ = 0.087
φ and ω scans	θ_max_ = 25.1°, θ_min_ = 1.5°
Absorption correction: multi-scan (*SADABS*; Sheldrick, 1996)	*h* = −11→7
*T*_min_ = 0.979, *T*_max_ = 0.982	*k* = −31→30
15630 measured reflections	*l* = −14→14

### Refinement

**Table:**

Refinement on *F*^2^	Primary atom site location: structure-invariant direct methods
Least-squares matrix: full	Secondary atom site location: difference Fourier map
*R*[*F*^2^ > 2σ(*F*^2^)] = 0.070	Hydrogen site location: inferred from neighbouring sites
*wR*(*F*^2^) = 0.215	H-atom parameters constrained
*S* = 1.02	*w* = 1/[σ^2^(*F*_o_^2^) + (0.0857*P*)^2^] where *P* = (*F*_o_^2^ + 2*F*_c_^2^)/3
5310 reflections	(Δ/σ)_max_ < 0.001
393 parameters	Δρ_max_ = 0.25 e Å^−^^3^
0 restraints	Δρ_min_ = −0.22 e Å^−^^3^

### Special details

**Table:**

Geometry. All esds (except the esd in the dihedral angle between two l.s. planes) are estimated using the full covariance matrix. The cell esds are taken into account individually in the estimation of esds in distances, angles and torsion angles; correlations between esds in cell parameters are only used when they are defined by crystal symmetry. An approximate (isotropic) treatment of cell esds is used for estimating esds involving l.s. planes.
Refinement. Refinement of F^2^ against ALL reflections. The weighted R-factor wR and goodness of fit S are based on F^2^, conventional R-factors R are based on F, with F set to zero for negative F^2^. The threshold expression of F^2^ > 2sigma(F^2^) is used only for calculating R-factors(gt) etc. and is not relevant to the choice of reflections for refinement. R-factors based on F^2^ are statistically about twice as large as those based on F, and R- factors based on ALL data will be even larger.

### Fractional atomic coordinates and isotropic or equivalent isotropic displacement parameters (Å^2^)

**Table:**

	*x*	*y*	*z*	*U*_iso_*/*U*_eq_	
C1	0.3223 (7)	0.6153 (3)	0.9041 (5)	0.133 (2)	
H1A	0.3370	0.6138	0.8294	0.199*	
H1B	0.3921	0.5940	0.9532	0.199*	
H1C	0.3348	0.6491	0.9313	0.199*	
C2	0.1753 (7)	0.5984 (2)	0.9018 (4)	0.103 (2)	
H2A	0.1604	0.5990	0.9774	0.124*	
H2B	0.1613	0.5644	0.8736	0.124*	
C3	0.0711 (7)	0.6969 (3)	0.9716 (6)	0.157 (3)	
H3A	0.0060	0.7223	0.9861	0.236*	
H3B	0.1598	0.7123	0.9635	0.236*	
H3C	0.0932	0.6737	1.0335	0.236*	
C4	0.0080 (7)	0.6728 (3)	0.8778 (5)	0.142 (3)	
H4A	−0.0179	0.6983	0.8199	0.170*	
H4B	−0.0835	0.6601	0.8892	0.170*	
C5	0.0391 (5)	0.62734 (19)	0.7115 (4)	0.0658 (13)	
C6	−0.0518 (5)	0.66052 (17)	0.6381 (4)	0.0682 (14)	
H6	−0.0908	0.6879	0.6675	0.082*	
C7	−0.0847 (4)	0.65369 (17)	0.5250 (4)	0.0619 (12)	
H7	−0.1472	0.6764	0.4792	0.074*	
C8	−0.0288 (4)	0.61409 (15)	0.4742 (3)	0.0472 (10)	
C9	0.0635 (4)	0.58217 (15)	0.5468 (3)	0.0501 (11)	
C10	0.0969 (5)	0.58814 (18)	0.6619 (4)	0.0651 (13)	
H10	0.1595	0.5654	0.7076	0.078*	
C11	0.1123 (4)	0.53531 (15)	0.3966 (3)	0.0490 (11)	
C12	0.1954 (5)	0.49728 (16)	0.3683 (4)	0.0580 (12)	
H12	0.2569	0.4789	0.4253	0.070*	
C13	0.1896 (4)	0.48581 (17)	0.2564 (4)	0.0562 (11)	
C14	0.3593 (6)	0.41531 (19)	0.3137 (4)	0.0833 (16)	
H14A	0.3737	0.3835	0.2797	0.100*	
H14B	0.3058	0.4088	0.3703	0.100*	
C15	0.5050 (6)	0.4366 (2)	0.3703 (5)	0.110 (2)	
H15A	0.5603	0.4420	0.3154	0.165*	
H15B	0.5563	0.4135	0.4265	0.165*	
H15C	0.4920	0.4678	0.4053	0.165*	
C16	0.3493 (8)	0.4691 (3)	0.0552 (6)	0.142 (3)	
H16A	0.3051	0.5018	0.0458	0.213*	
H16B	0.3526	0.4560	−0.0172	0.213*	
H16C	0.4468	0.4716	0.1023	0.213*	
C17	0.2632 (7)	0.4358 (2)	0.1076 (5)	0.1038 (19)	
H17A	0.2971	0.4017	0.1033	0.125*	
H17B	0.1619	0.4373	0.0650	0.125*	
C18	0.0957 (5)	0.51575 (18)	0.1741 (4)	0.0656 (13)	
H18	0.0884	0.5099	0.0980	0.079*	
C19	0.0150 (5)	0.55344 (17)	0.2056 (4)	0.0637 (13)	
H19	−0.0455	0.5726	0.1496	0.076*	
C20	0.0197 (4)	0.56410 (15)	0.3172 (3)	0.0471 (10)	
C21	−0.0665 (4)	0.60681 (15)	0.3484 (3)	0.0477 (10)	
C22	−0.2316 (4)	0.60356 (16)	0.2994 (3)	0.0493 (10)	
C23	−0.3254 (5)	0.56787 (17)	0.3198 (3)	0.0613 (12)	
H23	−0.2916	0.5398	0.3635	0.074*	
C24	−0.4729 (5)	0.57535 (19)	0.2726 (4)	0.0709 (14)	
H24	−0.5396	0.5519	0.2859	0.085*	
C25	−0.5239 (5)	0.6164 (2)	0.2065 (4)	0.0705 (14)	
H25	−0.6240	0.6206	0.1777	0.085*	
C26	−0.4286 (5)	0.65122 (17)	0.1826 (4)	0.0640 (13)	
H26	−0.4621	0.6785	0.1359	0.077*	
C27	−0.2806 (4)	0.64420 (15)	0.2306 (3)	0.0489 (11)	
C28	−0.1560 (4)	0.67624 (17)	0.2241 (4)	0.0535 (11)	
C29	0.1563 (5)	0.70297 (18)	0.2756 (4)	0.0663 (13)	
H29	0.0997	0.7142	0.2066	0.080*	
C30	0.3057 (5)	0.72331 (17)	0.3196 (4)	0.0587 (12)	
C31	0.3612 (6)	0.75637 (19)	0.2560 (4)	0.0783 (15)	
H31	0.3067	0.7650	0.1842	0.094*	
C32	0.4987 (6)	0.7771 (2)	0.2981 (5)	0.0872 (17)	
H32	0.5375	0.7990	0.2541	0.105*	
C33	0.5776 (5)	0.7650 (2)	0.4060 (5)	0.0733 (14)	
C34	0.5267 (5)	0.73148 (19)	0.4713 (4)	0.0706 (14)	
H34	0.5822	0.7228	0.5427	0.085*	
C35	0.3870 (5)	0.71047 (17)	0.4268 (4)	0.0649 (13)	
N1	0.0690 (5)	0.63236 (18)	0.8272 (4)	0.0940 (15)	
N2	0.2709 (4)	0.44784 (15)	0.2266 (3)	0.0749 (12)	
N3	−0.0361 (3)	0.65359 (12)	0.2909 (3)	0.0519 (9)	
N4	0.1058 (4)	0.67119 (13)	0.3293 (3)	0.0587 (10)	
O1	−0.1568 (3)	0.71531 (11)	0.1705 (3)	0.0705 (9)	
O2	0.7101 (4)	0.78780 (16)	0.4427 (3)	0.1002 (13)	
H2	0.7411	0.7832	0.5106	0.150*	
O3	0.3414 (4)	0.67909 (15)	0.4958 (3)	0.0928 (12)	
H3	0.2595	0.6687	0.4647	0.139*	
O4	0.1295 (3)	0.54163 (11)	0.5103 (2)	0.0666 (9)	

### Atomic displacement parameters (Å^2^)

**Table:**

	*U*^11^	*U*^22^	*U*^33^	*U*^12^	*U*^13^	*U*^23^
C1	0.120 (6)	0.175 (7)	0.096 (5)	0.019 (5)	0.013 (4)	−0.032 (5)
C2	0.098 (5)	0.142 (6)	0.069 (4)	−0.001 (4)	0.022 (4)	−0.030 (4)
C3	0.114 (6)	0.197 (8)	0.153 (7)	0.006 (5)	0.019 (5)	−0.095 (6)
C4	0.121 (5)	0.196 (8)	0.091 (5)	0.065 (5)	−0.009 (4)	−0.077 (5)
C5	0.049 (3)	0.086 (4)	0.056 (3)	0.009 (3)	0.000 (2)	−0.021 (3)
C6	0.054 (3)	0.072 (4)	0.074 (3)	0.012 (2)	0.006 (3)	−0.022 (3)
C7	0.055 (3)	0.058 (3)	0.064 (3)	0.013 (2)	−0.003 (2)	0.001 (2)
C8	0.038 (2)	0.048 (3)	0.050 (3)	−0.0026 (19)	0.001 (2)	0.000 (2)
C9	0.044 (3)	0.050 (3)	0.051 (3)	0.010 (2)	0.002 (2)	−0.004 (2)
C10	0.060 (3)	0.079 (4)	0.048 (3)	0.016 (2)	−0.002 (2)	−0.006 (3)
C11	0.055 (3)	0.050 (3)	0.038 (3)	0.001 (2)	0.003 (2)	0.002 (2)
C12	0.059 (3)	0.060 (3)	0.049 (3)	0.008 (2)	0.002 (2)	0.004 (2)
C13	0.051 (3)	0.058 (3)	0.057 (3)	0.001 (2)	0.009 (2)	−0.002 (2)
C14	0.096 (4)	0.068 (4)	0.090 (4)	0.023 (3)	0.032 (3)	−0.011 (3)
C15	0.078 (4)	0.108 (5)	0.139 (5)	0.017 (4)	0.018 (4)	0.001 (4)
C16	0.118 (6)	0.199 (8)	0.121 (6)	0.016 (5)	0.053 (5)	0.015 (5)
C17	0.114 (5)	0.110 (5)	0.093 (5)	0.028 (4)	0.037 (4)	−0.003 (4)
C18	0.068 (3)	0.080 (4)	0.045 (3)	0.013 (3)	0.008 (2)	0.000 (2)
C19	0.063 (3)	0.070 (3)	0.051 (3)	0.015 (2)	0.001 (2)	0.010 (2)
C20	0.045 (3)	0.051 (3)	0.043 (2)	0.000 (2)	0.006 (2)	0.006 (2)
C21	0.037 (2)	0.049 (3)	0.051 (3)	−0.0030 (19)	0.0008 (19)	0.004 (2)
C22	0.043 (3)	0.051 (3)	0.048 (2)	−0.006 (2)	0.000 (2)	−0.003 (2)
C23	0.047 (3)	0.068 (3)	0.062 (3)	−0.007 (2)	0.000 (2)	0.009 (2)
C24	0.054 (3)	0.078 (4)	0.075 (3)	−0.017 (2)	0.003 (3)	0.005 (3)
C25	0.040 (3)	0.087 (4)	0.076 (3)	−0.007 (3)	−0.003 (2)	0.006 (3)
C26	0.048 (3)	0.066 (3)	0.071 (3)	0.006 (2)	0.002 (2)	0.010 (3)
C27	0.039 (3)	0.049 (3)	0.052 (3)	−0.001 (2)	0.000 (2)	0.006 (2)
C28	0.051 (3)	0.051 (3)	0.055 (3)	0.003 (2)	0.006 (2)	0.004 (2)
C29	0.061 (3)	0.068 (4)	0.063 (3)	0.003 (3)	0.002 (3)	−0.003 (3)
C30	0.058 (3)	0.062 (3)	0.053 (3)	−0.003 (2)	0.007 (2)	−0.001 (2)
C31	0.087 (4)	0.085 (4)	0.066 (3)	−0.012 (3)	0.026 (3)	0.009 (3)
C32	0.079 (4)	0.102 (5)	0.081 (4)	−0.039 (3)	0.021 (3)	−0.010 (3)
C33	0.052 (3)	0.092 (4)	0.077 (4)	−0.024 (3)	0.018 (3)	−0.018 (3)
C34	0.054 (3)	0.084 (4)	0.071 (3)	−0.018 (3)	0.012 (3)	−0.009 (3)
C35	0.069 (3)	0.055 (3)	0.078 (4)	−0.009 (2)	0.032 (3)	0.005 (3)
N1	0.084 (3)	0.123 (4)	0.063 (3)	0.032 (3)	−0.004 (2)	−0.031 (3)
N2	0.083 (3)	0.080 (3)	0.061 (3)	0.027 (2)	0.016 (2)	−0.002 (2)
N3	0.037 (2)	0.051 (2)	0.062 (2)	−0.0056 (17)	0.0025 (17)	0.0110 (18)
N4	0.053 (2)	0.050 (2)	0.072 (3)	−0.0094 (18)	0.015 (2)	0.010 (2)
O1	0.058 (2)	0.061 (2)	0.084 (2)	0.0002 (15)	0.0007 (16)	0.0266 (18)
O2	0.070 (2)	0.137 (3)	0.092 (3)	−0.056 (2)	0.016 (2)	−0.017 (3)
O3	0.075 (3)	0.109 (3)	0.081 (2)	−0.028 (2)	−0.0046 (19)	0.027 (2)
O4	0.078 (2)	0.069 (2)	0.0468 (19)	0.0292 (17)	0.0047 (16)	0.0023 (16)

### Geometric parameters (Å, °)

**Table:**

C1—C2	1.453 (7)	C16—H16B	0.9600
C1—H1A	0.9600	C16—H16C	0.9600
C1—H1B	0.9600	C17—N2	1.476 (6)
C1—H1C	0.9600	C17—H17A	0.9700
C2—N1	1.486 (7)	C17—H17B	0.9700
C2—H2A	0.9700	C18—C19	1.374 (6)
C2—H2B	0.9700	C18—H18	0.9300
C3—C4	1.323 (7)	C19—C20	1.386 (5)
C3—H3A	0.9600	C19—H19	0.9300
C3—H3B	0.9600	C20—C21	1.504 (5)
C3—H3C	0.9600	C21—N3	1.496 (5)
C4—N1	1.434 (6)	C21—C22	1.527 (5)
C4—H4A	0.9700	C22—C23	1.366 (5)
C4—H4B	0.9700	C22—C27	1.381 (5)
C5—N1	1.380 (5)	C23—C24	1.384 (6)
C5—C10	1.388 (6)	C23—H23	0.9300
C5—C6	1.395 (6)	C24—C25	1.376 (6)
C6—C7	1.354 (5)	C24—H24	0.9300
C6—H6	0.9300	C25—C26	1.376 (6)
C7—C8	1.396 (5)	C25—H25	0.9300
C7—H7	0.9300	C26—C27	1.388 (5)
C8—C9	1.375 (5)	C26—H26	0.9300
C8—C21	1.504 (5)	C27—C28	1.473 (5)
C9—C10	1.375 (5)	C28—O1	1.231 (5)
C9—O4	1.377 (4)	C28—N3	1.362 (5)
C10—H10	0.9300	C29—N4	1.240 (5)
C11—C20	1.370 (5)	C29—C30	1.482 (6)
C11—O4	1.371 (4)	C29—H29	0.9300
C11—C12	1.380 (5)	C30—C31	1.365 (6)
C12—C13	1.391 (5)	C30—C35	1.389 (6)
C12—H12	0.9300	C31—C32	1.388 (6)
C13—N2	1.374 (5)	C31—H31	0.9300
C13—C18	1.413 (6)	C32—C33	1.385 (7)
C14—N2	1.465 (6)	C32—H32	0.9300
C14—C15	1.490 (7)	C33—O2	1.363 (5)
C14—H14A	0.9700	C33—C34	1.364 (6)
C14—H14B	0.9700	C34—C35	1.413 (6)
C15—H15A	0.9600	C34—H34	0.9300
C15—H15B	0.9600	C35—O3	1.335 (5)
C15—H15C	0.9600	N3—N4	1.388 (4)
C16—C17	1.458 (7)	O2—H2	0.8200
C16—H16A	0.9600	O3—H3	0.8200
			
C2—C1—H1A	109.5	N2—C17—H17B	108.8
C2—C1—H1B	109.5	H17A—C17—H17B	107.7
H1A—C1—H1B	109.5	C19—C18—C13	120.5 (4)
C2—C1—H1C	109.5	C19—C18—H18	119.8
H1A—C1—H1C	109.5	C13—C18—H18	119.8
H1B—C1—H1C	109.5	C18—C19—C20	122.9 (4)
C1—C2—N1	108.6 (5)	C18—C19—H19	118.5
C1—C2—H2A	110.0	C20—C19—H19	118.5
N1—C2—H2A	110.0	C11—C20—C19	116.3 (4)
C1—C2—H2B	110.0	C11—C20—C21	122.3 (4)
N1—C2—H2B	110.0	C19—C20—C21	121.3 (4)
H2A—C2—H2B	108.3	N3—C21—C8	110.5 (3)
C4—C3—H3A	109.5	N3—C21—C20	109.5 (3)
C4—C3—H3B	109.5	C8—C21—C20	110.6 (3)
H3A—C3—H3B	109.5	N3—C21—C22	99.2 (3)
C4—C3—H3C	109.5	C8—C21—C22	111.6 (3)
H3A—C3—H3C	109.5	C20—C21—C22	114.9 (3)
H3B—C3—H3C	109.5	C23—C22—C27	121.7 (4)
C3—C4—N1	126.8 (6)	C23—C22—C21	127.8 (4)
C3—C4—H4A	105.6	C27—C22—C21	110.5 (3)
N1—C4—H4A	105.6	C22—C23—C24	117.1 (4)
C3—C4—H4B	105.6	C22—C23—H23	121.5
N1—C4—H4B	105.6	C24—C23—H23	121.5
H4A—C4—H4B	106.1	C25—C24—C23	121.9 (4)
N1—C5—C10	121.3 (4)	C25—C24—H24	119.0
N1—C5—C6	122.4 (4)	C23—C24—H24	119.0
C10—C5—C6	116.3 (4)	C26—C25—C24	120.8 (4)
C7—C6—C5	121.4 (4)	C26—C25—H25	119.6
C7—C6—H6	119.3	C24—C25—H25	119.6
C5—C6—H6	119.3	C25—C26—C27	117.5 (4)
C6—C7—C8	122.9 (4)	C25—C26—H26	121.2
C6—C7—H7	118.6	C27—C26—H26	121.2
C8—C7—H7	118.6	C22—C27—C26	120.9 (4)
C9—C8—C7	115.5 (4)	C22—C27—C28	109.7 (4)
C9—C8—C21	122.2 (4)	C26—C27—C28	129.3 (4)
C7—C8—C21	122.4 (4)	O1—C28—N3	126.1 (4)
C8—C9—C10	122.5 (4)	O1—C28—C27	128.4 (4)
C8—C9—O4	122.8 (4)	N3—C28—C27	105.4 (4)
C10—C9—O4	114.6 (4)	N4—C29—C30	121.0 (4)
C9—C10—C5	121.4 (4)	N4—C29—H29	119.5
C9—C10—H10	119.3	C30—C29—H29	119.5
C5—C10—H10	119.3	C31—C30—C35	119.9 (4)
C20—C11—O4	123.0 (4)	C31—C30—C29	119.7 (4)
C20—C11—C12	122.4 (4)	C35—C30—C29	120.4 (4)
O4—C11—C12	114.5 (4)	C30—C31—C32	120.2 (5)
C11—C12—C13	121.6 (4)	C30—C31—H31	119.9
C11—C12—H12	119.2	C32—C31—H31	119.9
C13—C12—H12	119.2	C33—C32—C31	119.6 (5)
N2—C13—C12	122.4 (4)	C33—C32—H32	120.2
N2—C13—C18	121.4 (4)	C31—C32—H32	120.2
C12—C13—C18	116.2 (4)	O2—C33—C34	121.9 (5)
N2—C14—C15	114.0 (5)	O2—C33—C32	116.2 (5)
N2—C14—H14A	108.8	C34—C33—C32	121.9 (4)
C15—C14—H14A	108.8	C33—C34—C35	117.8 (4)
N2—C14—H14B	108.8	C33—C34—H34	121.1
C15—C14—H14B	108.8	C35—C34—H34	121.1
H14A—C14—H14B	107.7	O3—C35—C30	124.3 (4)
C14—C15—H15A	109.5	O3—C35—C34	115.0 (4)
C14—C15—H15B	109.5	C30—C35—C34	120.7 (4)
H15A—C15—H15B	109.5	C5—N1—C4	121.0 (5)
C14—C15—H15C	109.5	C5—N1—C2	120.4 (4)
H15A—C15—H15C	109.5	C4—N1—C2	118.4 (4)
H15B—C15—H15C	109.5	C13—N2—C14	119.9 (4)
C17—C16—H16A	109.5	C13—N2—C17	121.9 (4)
C17—C16—H16B	109.5	C14—N2—C17	117.9 (4)
H16A—C16—H16B	109.5	C28—N3—N4	129.9 (3)
C17—C16—H16C	109.5	C28—N3—C21	115.1 (3)
H16A—C16—H16C	109.5	N4—N3—C21	113.9 (3)
H16B—C16—H16C	109.5	C29—N4—N3	121.3 (4)
C16—C17—N2	114.0 (5)	C33—O2—H2	109.5
C16—C17—H17A	108.8	C35—O3—H3	109.5
N2—C17—H17A	108.8	C11—O4—C9	118.5 (3)
C16—C17—H17B	108.8		
			
N1—C5—C6—C7	−177.1 (4)	C25—C26—C27—C28	−176.9 (4)
C10—C5—C6—C7	1.8 (7)	C22—C27—C28—O1	179.4 (4)
C5—C6—C7—C8	−1.2 (7)	C26—C27—C28—O1	−3.2 (8)
C6—C7—C8—C9	−0.3 (6)	C22—C27—C28—N3	−0.6 (5)
C6—C7—C8—C21	179.8 (4)	C26—C27—C28—N3	176.8 (4)
C7—C8—C9—C10	1.1 (6)	N4—C29—C30—C31	176.7 (4)
C21—C8—C9—C10	−179.0 (4)	N4—C29—C30—C35	−5.8 (7)
C7—C8—C9—O4	−179.2 (4)	C35—C30—C31—C32	0.1 (7)
C21—C8—C9—O4	0.7 (6)	C29—C30—C31—C32	177.6 (5)
C8—C9—C10—C5	−0.4 (7)	C30—C31—C32—C33	−1.6 (8)
O4—C9—C10—C5	179.8 (4)	C31—C32—C33—O2	−178.3 (5)
N1—C5—C10—C9	177.9 (4)	C31—C32—C33—C34	2.7 (8)
C6—C5—C10—C9	−1.1 (7)	O2—C33—C34—C35	178.8 (4)
C20—C11—C12—C13	−0.2 (7)	C32—C33—C34—C35	−2.2 (8)
O4—C11—C12—C13	179.5 (4)	C31—C30—C35—O3	179.1 (5)
C11—C12—C13—N2	−179.5 (4)	C29—C30—C35—O3	1.6 (7)
C11—C12—C13—C18	0.9 (6)	C31—C30—C35—C34	0.3 (7)
N2—C13—C18—C19	179.8 (4)	C29—C30—C35—C34	−177.1 (4)
C12—C13—C18—C19	−0.7 (7)	C33—C34—C35—O3	−178.1 (4)
C13—C18—C19—C20	−0.3 (7)	C33—C34—C35—C30	0.7 (7)
O4—C11—C20—C19	179.6 (4)	C10—C5—N1—C4	−179.5 (5)
C12—C11—C20—C19	−0.8 (6)	C6—C5—N1—C4	−0.6 (8)
O4—C11—C20—C21	2.7 (6)	C10—C5—N1—C2	5.7 (7)
C12—C11—C20—C21	−177.6 (4)	C6—C5—N1—C2	−175.4 (5)
C18—C19—C20—C11	1.0 (6)	C3—C4—N1—C5	−145.1 (8)
C18—C19—C20—C21	177.9 (4)	C3—C4—N1—C2	29.7 (12)
C9—C8—C21—N3	−126.4 (4)	C1—C2—N1—C5	78.6 (6)
C7—C8—C21—N3	53.5 (5)	C1—C2—N1—C4	−96.3 (7)
C9—C8—C21—C20	−5.0 (5)	C12—C13—N2—C14	4.7 (7)
C7—C8—C21—C20	174.9 (4)	C18—C13—N2—C14	−175.8 (4)
C9—C8—C21—C22	124.3 (4)	C12—C13—N2—C17	179.7 (5)
C7—C8—C21—C22	−55.8 (5)	C18—C13—N2—C17	−0.8 (7)
C11—C20—C21—N3	125.3 (4)	C15—C14—N2—C13	−83.1 (6)
C19—C20—C21—N3	−51.4 (5)	C15—C14—N2—C17	101.7 (6)
C11—C20—C21—C8	3.4 (5)	C16—C17—N2—C13	79.7 (7)
C19—C20—C21—C8	−173.3 (4)	C16—C17—N2—C14	−105.3 (6)
C11—C20—C21—C22	−124.1 (4)	O1—C28—N3—N4	11.1 (7)
C19—C20—C21—C22	59.2 (5)	C27—C28—N3—N4	−168.9 (4)
N3—C21—C22—C23	179.7 (4)	O1—C28—N3—C21	178.8 (4)
C8—C21—C22—C23	−63.9 (6)	C27—C28—N3—C21	−1.2 (5)
C20—C21—C22—C23	63.1 (6)	C8—C21—N3—C28	−115.0 (4)
N3—C21—C22—C27	−2.5 (4)	C20—C21—N3—C28	122.9 (4)
C8—C21—C22—C27	113.9 (4)	C22—C21—N3—C28	2.2 (4)
C20—C21—C22—C27	−119.1 (4)	C8—C21—N3—N4	54.7 (4)
C27—C22—C23—C24	−2.9 (7)	C20—C21—N3—N4	−67.4 (4)
C21—C22—C23—C24	174.6 (4)	C22—C21—N3—N4	171.9 (3)
C22—C23—C24—C25	1.0 (7)	C30—C29—N4—N3	177.2 (4)
C23—C24—C25—C26	1.6 (8)	C28—N3—N4—C29	−27.2 (7)
C24—C25—C26—C27	−2.2 (7)	C21—N3—N4—C29	165.0 (4)
C23—C22—C27—C26	2.3 (7)	C20—C11—O4—C9	−7.5 (6)
C21—C22—C27—C26	−175.6 (4)	C12—C11—O4—C9	172.8 (4)
C23—C22—C27—C28	180.0 (4)	C8—C9—O4—C11	5.7 (6)
C21—C22—C27—C28	2.1 (5)	C10—C9—O4—C11	−174.5 (4)
C25—C26—C27—C22	0.3 (6)		

## References

[bb1] Bruker (1998). *SAINT* Bruker AXS Inc., Madison, Wisconsin, USA.

[bb3] Bruker (2005). *APEX2* Bruker AXS Inc., Madison, Wisconsin, USA.

[bb4] Deng, W.-J., Sun, D., Su, B.-Y., Wang, S.-P. & Zheng, H. (2009). *Acta Cryst.* E**65**, o1464. 10.1107/S1600536809020248 PMC2969317 21582767

[bb5] Kwon, J. Y., Jang, Y. J., Lee, Y. J., Kim, K. M., Seo, M. S., Nam, W. & Yoon, I. (2005). *J. Am. Chem. Soc.* **127**, 10107–10111. 10.1021/ja051075b 16011377

[bb6] Sheldrick, G. M. (1996). *SADABS* University of Göttingen, Germany.

[bb7] Sheldrick, G. M. (2008). *Acta Cryst.* A**64**, 112–122. 10.1107/S0108767307043930 18156677

[bb8] Tian, M.-Z. & Peng, X.-J. (2008). *Acta Cryst.* E**64**, o1645. 10.1107/S1600536808023611 PMC2962210 21203332

[bb9] Wu, D., Huang, W., Duan, C.-Y., Lin, Z.-H. & Meng, Q.-J. (2007). *Inorg. Chem.* **46**, 1538–1540. 10.1021/ic062274e 17266305

[bb10] Xu, Z.-H., Wang, H.-S., Tao, L.-T. & Wang, H.-W. (2009). *Acta Cryst.* E**65**, o1876. 10.1107/S1600536809025872 PMC2977299 21583570

[bb11] Zhang, L.-Z., Peng, X.-J., Gao, S. & Fan, J.-L. (2008). *Acta Cryst.* E**64**, o403. 10.1107/S1600536807068742 PMC2960358 21201431

